# Supplementary material for: Are carbon emissions trading and green financial instruments synergistic? -Comprehensive quantitative research based on content analysis
Source: PLoS One. 2024 Mar 7;19(3):e0298601. doi: 10.1371/journal.pone.0298601 (PMC10919601; doi:10.1371/journal.pone.0298601)
Supplement: S1 Data — (ZIP) [file pone.0298601.s001.zip › Data/地级市Z_F工作报告.zip!/╣≤╓▌/╫±╥σ╩╨/└┤╫╘╣1⁄2╓┌║┼í╛┬φ┐╦╩2╛▌═°í┐_2017.docx]

各位代表：

　　现在，我代表市人民政府向大会作工作报告，请予审议，并请各位政协委员和其他列席人员提出意见。

　　过去五年工作回顾

　　过去五年，在省委、省政府和市委的坚强领导下，在市人大、市政协的监督支持下，全市上下深入贯彻以习近平同志为核心的党中央治国理政新理念新思想新战略，主动引领经济发展新常态，坚守发展和生态两条底线，坚定不移实施主基调主战略和两大战略行动，圆满完成四届人大一次会议确定的各项目标任务，经济社会发展实现新跨越，遵义故事精彩纷呈、遵义形象熠熠生辉、遵义未来憧憬满怀。

　　一、赶超之力驰而不息，经济实力迈上新台阶。初步统计，2016年完成地区生产总值2403.9亿元，年均增长14%，经济总量实现五年翻番，从全国非省会地级城市第104位、西部非省会同类城市第14位分别跃升至第61位、第5位；人均地区生产总值38709元；三次产业结构调整为15.4︰44.2︰40.4；规模工业增加值突破千亿元，达1062.9亿元，年均增长13.5%；500万元以上固定资产投资2068.9亿元，全社会固定资产投资累计突破万亿元，年均增长35%以上；财政总收入480.1亿元，年均增长18.9%；一般公共预算收入187.5亿元，年均增长17.2%；社会消费品零售总额723.4亿元，年均增长15.6%；城镇居民人均可支配收入27097元，年均增长9.2%；农村居民人均可支配收入突破万元，达10109元，年均增长14.1%；金融机构存贷款余额4305.8亿元、2264.9亿元，年均分别增长25.1%、26.1%。全面小康实现程度89%，五年提升21个百分点，5个区（市）率先通过全面小康达标验收。各县（市、区）地区生产总值实现翻番，县域经济比重达60%，仁怀市跻身全国百强县。

　　二、转型之战攻坚突围，结构调整实现新跨越。新型工业化加速突破。工业经济总量稳居全省第一，规模工业企业突破1000家。县县搭起经济开发区或工业园区平台，建成标准厂房952万平方米、入驻企业786户。大数据、新能源汽车、吉他等新兴产业从无到有、迅猛发展，茶酒“姊妹篇”、装备制造等传统产业转型升级、稳步发展。绿色城镇化加速崛起。常住人口城镇化率达47%。遵义县撤县设区，南部新区挂牌运行，打通高新快线、官井隧道、东南大道、九汇大道等骨干道路，形成以五区为支撑的200万人口城市框架。所有县城建成区面积实现倍增，创建国家卫生县城4个、省级文明县城9个。建成茅台、土城等56个特色示范小城镇。“四在农家·美丽乡村”创建覆盖226个乡镇1700多个村，90%以上农村人口受益。农业现代化加速推进。农业增加值实现五年翻番，五大主导产业规模壮大，建成省级现代高效农业园区52个，培育省级以上龙头企业128家、市级龙头企业555家、各类农民专业合作社5945个、家庭农场550个，认证“三品一标”（1）农产品211个，粮经比由6︰4调整为4︰6，农业科技贡献率达53%。旅游产业化加速提升。累计接待游客2.75亿人次、实现综合收入2400亿元，年均分别增长35%。海龙屯土司遗址成功申遗。推出遵义会议纪念体系、赤水绿道、茅台古镇、双河溶洞等旅游品牌和花茂、杉坪等农旅融合示范点，建成市游客集散中心和一批星级酒店、精品酒庄茶庄等服务设施，新增国家4A级景区11个。现代服务业加速发展。电子商务交易额突破300亿元。银行业、保险业金融机构分别达47家、25家，全市首家互联网金融资产交易中心上线运营。遵义国际商贸城、新雪域冷链物流等项目建成运营，限额以上商贸企业达827家。

　　三、瓶颈之困不断破解，基础设施取得新突破。交通条件显著改善。遵义新舟机场旅客年吞吐量突破110万人次，茅台机场即将建成。渝黔快铁加快建设。高速公路和一级公路通车里程突破1000公里，实现县县通高速。新增国省干线3200公里、二级公路1400多公里，农村公路通车里程达2.6万公里，基本实现村村通油（砼）路、乡乡有客运站、建制村通客运。建成航运码头17个。水利保障能力增强。建设骨干水源工程56座，建成城镇供水厂222座，新增供水能力8亿立方米。治理病险水库186座、中小河流71条，发展灌溉面积67.6万亩。400万农村群众喝上安全饮用水。能源通信基础夯实。页岩气勘探取得重大进展。一批火电、水电、风电项目建成发电，电力总装机突破1000万千瓦，农村电网改造全面完成。中卫—贵阳（遵义段）和遵义至习水天然气支线管道全线贯通。与贵阳、安顺实现通信同城化，在全省率先实现移动4G信号乡乡通，中心城区免费WiFi覆盖面达20平方公里。

　　四、改革之策破立并举，开放开发开创新局面。重点改革成果丰硕。政府职能不断转变，市级非行政许可审批事项全部取消，累计下放市级行政审批事项183项。62项国家级、省级改革试点深入推进，赤水河流域生态文明制度改革经验在全省全国推广，湄潭县农村集体经营性建设用地入市改革进一步深化，率先在全省实施公共资源交易改革。创新活力持续涌现。建成院士工作站及重点实验室47个、国家高新技术企业84个、省级以上企业技术中心44个，实施国家和省部级科技成果转化600多项，建成白酒、茶叶、低压电器产品、高压分接开关国家级检测中心，获得国家授权专利7200件，综合科技进步水平指数达69.5%。注册商标1.7万件，新增驰名商标14件。对外开放步伐加快。“六大开放平台”（2）加速搭建，遵义经济技术开发区获批长江经济带国家级转型升级示范开发区，遵义新舟机场开通临时航空口岸。承接上海对口帮扶、主动融入成渝成效明显。“5个100工程”（3）吸纳企业3000多家。市场主体近38万户，民营经济比重达55%。招商引资累计到位资金9000多亿元。累计完成外贸进出口总额40亿美元、利用外资8.5亿美元，年均分别增长30%、40%。

　　五、绿色之路方兴未艾，生态文明开启新时代。成功创建国家环境保护模范城市。建成国家生态示范区5个，湄潭县、赤水市在全省率先成为国家生态县（市）。绿化造林400万亩，治理石漠化面积753平方公里、水土流失面积1850平方公里，森林覆盖率达57%。淘汰落后产能533万吨。建成55座集镇污水处理厂，城镇污水处理率90%，赤水河流域乡镇污水处理设施实现全覆盖。城乡生活垃圾无害化处理率85%。城市（县城）环境空气质量优良率达95%。主要河流监测断面达标率90.9%。县城以上集中式饮用水源地水质达标率100%。

　　六、为民之心矢志不渝，社会事业赢得新发展。贫困人口大幅减少。累计减少农村贫困人口109.47万人，贫困发生率从21.73%下降到4.88%。预计4个贫困县按国标脱贫退出、116个贫困乡镇全部按省标脱贫退出、416个贫困村按国标出列。教育事业优先发展。新（改、扩）建各类基础教育项目5996个，41所学校列入省级以上示范校，6个县（市、区）通过国家义务教育基本均衡验收，15个县（市、区）通过省级“普十五”验收，5所院校7万师生入驻遵义大学城，贵州大学茶学院落户湄潭。就业创业有效推进。新增城镇就业54.4万人，转移农村劳动力60.2万人，城镇登记失业率控制在4.2%以内，荣获全国创业先进城市称号。公共服务稳步提升。人口计生工作扎实有效。公共卫生服务体系加快完善，市县公立医院全部实现“二甲”以上目标，城乡居民医保参保率保持在98%以上。在全省率先建成国家公共文化服务体系示范区。“一场四馆”（4）县县全覆盖。社会保障力度加大。城乡居民养老、医疗、就业等保障体系日益健全，城乡低保应保尽保，乡乡建起敬老院，建成保障性安居工程29万套，改造农村危房22万户，实施移民搬迁14.19万人。社会治理全面加强。安全生产形势持续向好，社会治安防控、矛盾纠纷排查化解力度加大，连续第3次蝉联“长安杯”，连续3年进入“中国最安全城市排行榜”，人民群众安全感跃居全省前列。外事侨务、民族宗教、国防教育、人民防空、防灾减灾、档案管理、妇女儿童、残疾人、地方志、红十字等事业取得新发展。

　　七、勤廉之风植根黔北，政府效能得到新提升。深入开展党的群众路线教育实践活动和“三严三实”（5）专题教育、“两学一做”（6）学习教育，持续推进解放思想“十破十立”（7）和“五梳理三带头”（8），在抗击自然灾害、决战脱贫攻坚等各个方面涌现出大批先进典型。始终把纪律和规矩挺在前面，深入推进党风廉政建设，强化民生监督，坚决整治“四风”，底线守得更牢，笼子扎得更紧。深入抓好机关效能建设，大力推行清单管理，落实“三集中、三到位”（9），竞相迸发出敢于担当、主动作为、比学赶超、狠抓落实的强大合力。

　　刚刚过去的2016年，是“十三五”开局之年，省委对遵义作出了“坚持红色传承，推动绿色发展，奋力打造西部内陆开放新高地”的战略定位。一年来，我们以纪念红军长征胜利80周年为契机，保持“开局就是决战、起步就是冲刺”的精神状态，坚定信心，砥砺奋进，增比进位综合测评位列全省第二，“十三五”旗开得胜、强势起飞。坚持以脱贫攻坚为统揽，全力确保首战告捷，实施扶贫项目947个，预计减少贫困人口23.29万人，完成易地脱贫搬迁72339人，创建48个市级脱贫攻坚示范点，贫困村发生率下降20.59个百分点，在全省遥遥领先。坚持以供给侧结构性改革为主线，加大各领域有效供给，5家企业在新三板上市，对135个项目实施技术改造，销售商品住房972万平方米，营改增降税减负23.6亿元，降低企业用电成本4亿元，完成基础设施和民生事业投资400亿元。坚持以大数据为引领，大力培育新动能，新引进智能终端制造企业98家，生产手机1.15亿台，占全省98%以上，位居全国第4，生产电脑300多万台、占全省96%以上，大数据产业规模总量突破800亿元。坚持以四个建设年活动为载体，集中开工项目1515个，建成投产项目110个，组织承办省市项目观摩活动6次，新增限上商业企业103家、外贸企业82家、外资企业10家，招商引资到位资金2288亿元、增长16.7%。坚持以创建全国文明城市为目标，大力实施“十大工程”（10），遵龙、凤新等骨干道路加快建设，中心城区15所新（迁）建中小学、市一医内科综合大楼建成投用。中心城区绿地率、绿化覆盖率、人均公共绿地面积居全省第一。坚持以办节办会为工作抓手，举办了“茶博会”、全省第十一届旅游产业发展大会、两届全市“旅发会”和首届“辣博会”、市运会等活动，扩大了遵义影响力，展示了遵义新形象，唱响了遵义好声音。

　　各位代表!五年发展实践告诉我们，遵义是一片人文厚重的沃土，更是充满希望的热土。站在新的发展起点，我们必须解放思想、主动作为，坚决贯彻稳中求进工作总基调，牢固树立创新思维、精准思维、底线思维、法治思维，顺应大数据时代，迎接大众旅游时代，走向生态文明新时代，彻底摆脱思想贫困。必须生态优先、绿色发展，牢牢守住发展和生态两条底线，既要绿水青山又要金山银山，实现两条底线一起守、两个成果一起收。必须弯道取直、后发赶超，以供给侧结构性改革为主线，全面深耕改革、力推创新、加快开放，走出一条速度快、质量高、效益好、百姓富、生态美的转型发展之路。必须以人为本、全民共享，为民服务决不一阵风，始终把群众安全感、获得感、幸福感和满意度作为谋划和检验工作的根本标准，千方百计提高人民群众生活水平。必须依法行政、从严治政，牢固树立“四个意识”（11），把忠诚与担当写在黔北大地上，艰苦奋斗、长期奋斗、不懈奋斗，努力把遵义建设得更加美好。

　　各位代表，这些成绩和经验的取得，得益于党中央、国务院的亲切关怀，得益于省委、省政府和市委的坚强领导，得益于市人大、市政协的监督支持，得益于全市干部群众的艰苦奋斗，得益于社会各界的倾力相助和上海等城市的对口帮扶。在此，我谨代表市人民政府，向全市各族人民、各级干部致以崇高的敬意！向各民主党派、工商联、人民团体、无党派人士，向驻遵人民解放军、武警消防官兵和民兵预备役人员、公安干警，向历届老领导老同志，向所有关心支持遵义经济社会发展的各界人士，表示衷心的感谢！

　　在充分肯定成绩的同时，我们也清醒地看到，全市最鲜明的特征是贫穷落后、最突出的短板是基础设施、最根本的症结是开放度不高，表现在经济总量小、人均水平低、传统产业转型慢、新兴产业还不强、新经济新业态还较脆弱、贫困面宽量广程度深、民生短板多欠账大、改革深度广度自觉度不够、科技金融和人才创新等要素欠缺，思想贫困和本领恐慌问题依然突出。我们将坚持问题导向，把研究问题破解难题竞赛进行到底。

　　今后五年奋斗目标及战略任务

　　未来五年，贫穷落后仍是遵义的主要矛盾，加快发展仍是遵义的根本任务。我们必须抢抓一切机遇、付出一切努力、克服一切困难，不忘初心，继续前进，全面向贫困宣战、向小康进军、向开放进发，“撸起袖子加油干，就一定能够走好我们这一代人的长征路”，把遵义建设成为红色传承的精神高地、绿色发展的战略高地、西部内陆开放新高地。

　　今后五年工作的总体要求是：高举中国特色社会主义伟大旗帜，以邓小平理论、“三个代表”重要思想、科学发展观为指导，深入学习贯彻习近平总书记系列重要讲话精神，牢固树立“四个意识”特别是核心意识、看齐意识，统筹推进“五位一体”总体布局，协调推进“四个全面”战略布局，全面落实“五大新发展”理念，按照省委、省政府和市第五次党代会决策部署，以守牢底线、走好新路、奔快小康为总纲，坚持红色传承，推动绿色发展，坚定不移实施主基调主战略，深入实施“五大行动”，全力构建“五大体系”，全力摆脱贫困，决胜全面小康，奋力打造西部内陆开放新高地。

　　主要预期目标是：地区生产总值年均增长13%左右，在2015年基础上翻一番，跃居西部非省会同类城市前3位；人均生产总值突破1万美元；三次产业结构调整为 10︰46︰44；500万元以上固定资产投资年均增长20%以上；规模工业增加值年均增长13%以上；社会消费品零售总额年均增长13.5%；一般公共预算收入年均增长12%以上；城乡居民人均可支配收入年均增长13%、13.5%；确保2019年全面稳定脱贫，2020年与全国同步建成全面小康社会。

　　一、深入实施五大行动，坚决如期摆脱贫困实现全面小康

　　深入实施大扶贫战略行动，打造具有全国示范性的脱贫攻坚遵义模式。全方位精准贫困人口。落实“八个精准”（12），推进“五个一批”（13），扎实抓好“十项行动”（14），对建档立卡贫困人口实行智能化动态管理，确保2017年习水县，2018年正安县、道真自治县、务川自治县按国家标准脱贫退出；2019年所有贫困村出列，所有建档立卡贫困人口稳定脱贫；2020年以县为单位实现全面小康。全领域撕掉贫困标签。紧扣“两率四性”（15），易地扶贫搬迁4.5万户18万人，实现乡乡有产业扶贫基地、村村有特色主导产品、户户贫困家庭至少1人稳定就业，培育壮大村集体经济。建成通村油（砼）路3000公里以上，全面解决农村人口饮水安全问题，完成农村幼儿园、寄宿制学校标准化建设，落实贫困家庭学生和医疗救助扶贫对象资助政策，实现低保和扶贫标准“两线合一”。深化沪遵合作。发挥资本市场作用服务脱贫攻坚。深入开展示范创建和“千企帮村、万户结对”等活动。全过程落实精准脱贫责任。强化“五主”责任（16），落实最严格的差异化考核和常态化督促检查机制，高度重视和防范工作不精准、执行政策跑偏走调、易地脱贫搬迁稳不住等问题，切实防止急功近利，坚决制止弄虚作假，绝不允许数字脱贫，确保脱贫成果真实可信、社会认可、百姓认账。

　　深入实施大数据战略行动，打造大数据时代西部前沿城市。建成国内知名的智能终端产业集聚区。顺应“万物互联、智能时代”趋势，大力培育大数据核心业态、关联业态和衍生业态。以智能终端产品制造为主攻方向，聚焦“321”目标（17），实施“五个计划”“六大工程”（18），力促电子信息制造业倍增，引进培育智能终端及配套企业300家以上，形成一批满足大数据应用需求的产品和系统，大数据信息产业规模总量突破4000亿元。建成国内领先的大数据应用示范基地。深入推进“互联网+”行动计划，促进大数据在各领域广泛应用、深度融合。加快政府数据“聚通用”,着力消除“信息孤岛”“数据烟囱”,实现政府系统数据互联共享。实施大数据应用示范和软件与信息技术服务业培育工程，加快智慧城市建设，建成市级数据中心、城市公共基础信息云平台、国家智慧水务云＆端产业基地和各类行业云等公共服务应用平台，建成服务外包呼叫中心10万座席左右。建成国内一流的信息基础设施体系。围绕骨干网络优化、城镇网络覆盖、农村宽带延伸和信息惠民、信息安全，实施“无线网络·满格遵义”、宽带乡村、物联网、5G网络、免费WiFi等信息工程，加快下一代互联网建设，建成全光网城市，构建高速、移动、安全、泛在的新一代信息基础设施体系。

　　深入实施大生态行动，在国家生态文明试验区建设中当先锋。与时俱进创新绿色制度。促进环境保护地方立法，实行最严格的耕地保护、环境保护制度，严格落实《遵义市市辖区集中式饮用水水源保护条例》。全面推广赤水河流域改革成果，全面推行“河长制”，完善生态文明考核评价、自然资源资产产权、国土空间开发等制度，继续在全省全国生态文明体制机制改革中走前列作示范。绵绵用力筑牢绿色屏障。强化主体功能区规划落实，深入推进“月月造林”“增色添彩”行动，治理石漠化面积333平方公里、水土流失面积1500平方公里，建成100个山体公园、180个森林公园（游园）、150个湿地公园，对25度以上和重要水源地15度以上坡耕地全部退耕还林还草，森林覆盖率达62%以上，努力把遵义建成公园市。实施“治污治水·洁净家园”五年攻坚行动，彻底整治乌江、赤水河等流域环境，推进农村环境集中连片治理，实现城镇污水处理率95%以上、城乡生活垃圾无害化处理率94%以上、城市（县城）环境空气质量优良率98%以上、县城以上集中式饮用水源水质达标率100%。久久为功培育绿色文化。坚持“醉美遵义·拒绝污染”，大力推进循环低碳发展。全面推进生态创建，确保2020年建成国家生态市。积极倡导勤俭节约、绿色低碳、文明健康的生活方式和消费模式，创新开展“生态日”活动，让黔北大地处处充满绿色脉动。

　　深入实施大旅游行动，建成首批国家全域旅游示范区。倾力打造旅游精品。实施精品景区、精品线路建设工程，打造全国一流的山地特色旅游产品体系和“一心一圈三线”（19）精品旅游线路，建成国家5A级景区2个以上、国家4A级景区15个以上。全面构建全域旅游发展新格局，大幅提升省外境外游客比例，游客接待人次和综合收入年均分别增长35%以上。倾力完善服务配套。加强旅游市场和服务质量体系建设，建成一批旅游交通和游客集散中心、数据中心、星级酒店、旅游公厕等服务设施，新增接待床位5万张以上，高标准建设具有国际品牌竞争力的精品酒庄、精品茶庄和精品驿站100个以上，建立覆盖国内外主要客源的旅游营销和配套服务体系。倾力推进融合示范。建成一批特色工旅、农旅、文旅等融合发展示范基地、示范小镇和示范企业，培育和引进集团化、品牌化大型旅游企业，大力开发避暑养生、休闲养老、温泉度假、汽车露营、科普探险、研学影视等多种旅游新业态新产品，全面提升旅游产业规模化、精品化、市场化、国际化水平。

　　深入实施大健康行动，跻身全国健康城市行列。基本建立健康产业体系。大力发展康养旅游产业，实施“3+3+N”（20）行动计划，建成国际温泉康养旅游目的地；打造贵州遵义桃花江国际健康医疗旅游示范基地，建设省级健康养生产业示范基地10个以上，建成覆盖全生命周期、全地域发展的休闲养生目的地。大力发展智慧健康产业，培育远程诊疗、健康检测、智能医院等新业态，实现市县乡远程诊疗网络全覆盖。大力发展健康药食材产业，加快深溪、道真等药业园区建设，培育10个在全国有较大影响的优势中药材品种、3个以上中药材种植大县、2个以上产值达10亿元的健康饮用水生产企业。大力发展健康运动产业，打造一批国家级山地户外运动基地和国际山地户外精品赛事。全力补齐医疗卫生短板。始终把人民健康放在优先发展战略地位，实施医疗卫生“五大工程”（21），迁建市中医院，新增10家三级医院，建设20个社区卫生服务中心、50个乡镇卫生院、500个村卫生室、14个疾控中心规范化达标项目。加强食品药品安全监管和医疗卫生人才队伍建设。坚持计划生育基本国策。疾病防控、妇幼保健、卫生监督、卫生应急等专业公共卫生服务达到国家标准。全面深化医药卫生体制改革。建立现代医院管理制度，破除公立医院逐利机制，健全“三医联动”（22）和“三重保障”（23）体系，基本建立覆盖城乡居民的基本医疗卫生制度，人人享有基本医疗卫生服务。实施健康细胞工程，建成一批健康乡镇、健康社区、健康单位、健康家庭，全力建设健康遵义。

　　二、全面提高改革开放水平，打造西部内陆开放新高地

　　构建便捷高效的开放通道。全面构建参与国内区域合作、面向国际开放的通道体系。全方位承接上海对口帮扶。积极承接产业、资金、技术、人才辐射，建成东西部产业合作示范区，借上海之“船”融入长江经济带和海上丝绸之路。全方位参与成渝市场竞争。加快与成渝地区实现“四个互联互通”（24），规划建设渝黔快铁经济带，积极融入中新（重庆）战略性合作互联互通示范项目平台，借成渝之“梯”融入丝绸之路经济带。全方位与贵阳互联互动。建成兰海高速重遵、贵遵扩容工程，推动贵遵城市间公共服务一体化，借贵阳之“台”融入珠三角，连接“中国—中南半岛”经济走廊。

　　构筑面向全球的开放平台。建成六大引领性平台。推动遵义经济技术开发区跻身全国50强、西部10强。遵义综合保税区核心功能增强，引进培育企业300家以上。建成遵义新舟机场航空口岸，大力申建陆路口岸、国家肉类和水果指定进境口岸、国际邮政口岸。建成遵义国家高新区，力争实现千亿级。发挥遵义大学城集聚效应，建设一批低成本、便利化、开放式众创空间和虚拟创新社区，做实做强遵义软件园和大学生创业园。建成一批交流性平台。加快融入成渝的物流高速路网和信息高速路网建设，力争建成10个共建园区和产业承接基地、10个商务和信息交流平台。高水平办好“茶博会”“辣博会”“酒文化节”“赤水河国际山地旅游节”等活动，打造2个以上国际性展会平台。建成一批集聚性平台。深入推进“5个100工程”和“1351”产业园区成长计划（25），全力打造“绿智园区”，全面提升17个省级产业园区集聚水平和配套能力，新建标准厂房500万平方米，建成2个1000亿级、3个500亿级、5个300亿级、9个100亿级园区，推动仁怀、新蒲开发区申建国家级开发区。建成一批创新性平台。实施科技创新“五大行动”（26），新建市级以上工程技术研究中心、企业技术中心、重点实验室50个以上，科技创新平台达200个，科技型企业达500家，新增发明专利申请量5000件。

　　培育竞争力强的开放主体。全力以赴招大引强。引进世界500强、国内500强和行业龙头企业，招商引资到位资金年均增长15%以上。蓬勃发展民营经济。全面深化商事制度改革，打造“遵商联盟”品牌，各类市场主体实现翻番，民营经济比重达60%以上。培育一批年主营业务收入超百亿元的龙头企业、超十亿元的小巨人企业和高成长性企业，新增规模工业企业800家。培育壮大外向企业。外资企业突破100家、外贸企业突破1000家，外贸进出口总额和实际利用外资年均分别增长20%以上。全力推动企业上市。力争50家企业在新三板挂牌，24家企业在主板、中小板或创业板上市。

　　打造西部一流的开放环境。坚持向改革要动力。更好地发挥经济体制改革的牵引作用，力推有利于增添经济发展动力、有利于促进社会公平正义、有利于增强人民群众获得感、有利于调动广大干部群众积极性的改革，坚决砍掉约束创造力、阻碍生产力的羁绊，让各类市场主体活力竞相涌流。坚持向创新要活力。全面推进“大众创业，万众创新”，高端嫁接、引进具有现实生产力转化能力的省外科技领军人才。实施质量提升、品牌带动和标准引领行动计划，力争制定一批国家、行业和地方标准，新增中国驰名商标10个以上、国家地理标志保护产品30个、贵州省著名商标和名牌产品各100个。全社会科研经费支出占GDP比重达2%，综合科技进步水平指数达70%以上，进入国家创新型城市行列。坚持向服务要效率。进一步转变政府职能，打造“五全”服务（27）网络，构建“亲”“清”新型政商关系。深入推进“法治遵义”“诚信遵义”建设，广泛开展“百万市民学普通话、学外语”活动，培育兼容并包的开放文化，厚植全民化、国际化无障碍开放交流的思想土壤。开放程度达80%以上。

　　三、全面加快产业转型升级，构建现代特色产业体系

　　坚定不移做强新型工业。建设三大基地（28）、四大集聚区（29），建成四个百亿级、五大千亿级产业（30），做大做靓六张名片（31）。做强茶酒等传统产业。围绕“一看三打造”（32）目标，强化品牌打造、市场开拓、转型升级和融资服务，培育壮大行业领军企业，加强线上线下营销创新，实现白酒产量60万千升，唱响“中国酱香·赤水河谷”地域品牌。围绕打造“中国茶叶第一市”目标，全面提升“遵义红”“遵义绿”、正安白茶和“东有龙井·西有凤冈”品牌效应，实现茶产业综合产值250亿元。围绕退城进园、兼并重组、技术改造、产品升级、业态创新，支持有市场前景的能矿、原材料、化工等产业转型升级。打造新能源产业集群。大力发展智能电动汽车制造和互联网汽车、汽车物联网，建成新能源汽车产业园，形成在全国有影响力的新能源汽车产业集群。加快页岩气勘探、利用和产业化步伐，打造页岩气勘探开发、综合利用全价值链产业集群。推进水电、风电和煤层气、生物质能等清洁能源开发利用。抢占军民融合制高点。积极融入“贵遵安”军民融合产业示范带，加快贵州航天高新技术产业园建设，重点发展航天配套设备、航空航天零部件和工程基础件，大力促进“军转民”，积极培育“民参军”，军民融合产业产值突破100亿元。

　　坚定不移做优现代山地特色高效农业。深入实施“1234”战略（33），大力推进资源集合、要素聚合、产业融合、模式整合，加快五大主导产业（34）规模化集群化发展，建成150个县级以上现代高效农业示范园区，实现农业“接二连三”。深化农业供给侧结构性改革，大力发展农业先进生产力和生产方式，建设现代农业庄园，引导流转土地300万亩，培育农民专业合作示范社500个、家庭农场2000个，省级以上农业产业化经营龙头企业达150家，培养5万名新型职业农民。加快农业标准化循环化发展，健全完善农业科技和社会化服务体系、农产品质量安全追溯和监管体系、农产品销售和物流体系，创建2个国家级农产品质量安全县、2个有机农产品认证示范区，建成全国无公害绿色有机农产品供应基地。“三品一标”农产品认证比例达60%以上。保障粮食安全，建设高标准农田100万亩，稳定发展10万亩以上城市保供蔬菜基地。着力打造全国第一的方竹笋产业基地。

　　坚定不移做大现代服务业。实施现代服务业“十百千”工程（35），加快以中央商务区为引领的现代服务业集聚区建设。大力完善物流网络，建成6个现代物流集聚区，培育3A级以上物流企业8家。加快城乡电子商务综合平台建设，培育电商骨干企业200家，电商交易额年均增长25%以上。推进金融、知识、科技、人力资本密集的生产性服务业发展，大力培育数字经济、总部经济、商业咨询等新业态，力争银行业金融机构达50家，建成新蒲金融商务会展集聚区，整合壮大一批会计师事务所，新增律师事务所25家、物业管理机构60家。提升房地产、文化娱乐、家政服务、教育培训、影视传媒等发展水平，运用现代信息技术改造提升传统商贸业，发展精细化、多元化生活性服务业。

　　四、全面统筹城乡区域发展，构建现代山地城镇体系

　　加快山地特色新型城镇化。坚持产城景一体、铁公机联动、山水田融合、村社园统筹、文教医配套，彰显“民国风”建筑元素，有序推进农业转移人口市民化，户籍人口和常住人口城镇化率分别达45%、57%。全力打造山水城市。坚持中心城区带动，构建以五个主城区为核心，仁怀市、桐梓县、湄潭县、绥阳县为支撑的遵义都市圈，建成200万人口规模的区域性中心城市。巩固提升县城倍增计划，全面增强县城综合承载能力，吸纳人口150万以上。加快绿色建筑与装配式建筑发展。推进“海绵城市”“公交都市”建设，完善城区立体交通网络、公共停车场、地下综合管廊、物流货运中心等基础设施。建立权责明晰、服务为先、管理优化、执法规范、安全有序的城市管理体制。全力打造特色小镇。突出“六型示范”（36），精心打造100个“六型”特色示范小城镇，全市小城镇吸纳人口100万以上。全力打造美丽乡村。突出黔北民居“七要素”（37），深入实施小康“6+1”行动计划（38），集中力量打造600个各具特色的美丽乡村示范点，2018年实现集中居住村寨创建全覆盖，2020年农户受益全覆盖。全力打造和谐社区。以“智慧社区”为引领，打造服务多元、功能完善、管理有序、文明祥和的新型社区，建成全国和谐社区示范市。

　　加快四大区域差异化发展。强化“四大区域”精准指导，推进中部跨越、西部转型、东部崛起、北部突破，确保经济总量县县翻番、全部过百亿。中部排头兵引领示范。坚持高点定位、高端引领，统筹推进中心城区建设，建设智能终端制造和新能源汽车产业集聚区、现代服务业集聚区、军民融合创新示范区，实现经济总量、产业层次、开放水平、城市品位重大跨越，地区生产总值达2500亿元以上。西部领头雁奋起直飞。坚持产业提升、创新引领，全面推进“四河四带”（39）建设，着力打造赤水河流域生态经济示范区和特色产业创新示范区，实现发展绿色化、经济循环化、业态新型化的重大转型，地区生产总值达1600亿元以上。东部先遣队奋力挺进长三角。坚持区域一体、生态引领，注重培育生态经济和新型高端业态，建设“四在农家·美丽乡村”转型发展示范区、统筹城乡发展试验区、高端休闲度假养生体验区，实现生态产业化、产业生态化的快速崛起，地区生产总值达500亿元以上。北部桥头堡率先融入成渝。坚持基础先行、小康引领，加快区域特色优势产业发展，全力突破基础设施瓶颈，打造脱贫攻坚示范区、特色农产品集聚区、民族文化旅游体验区、渝遵开放合作创新区，实现脱贫攻坚、后发赶超、同步小康重大突破，地区生产总值达400亿元以上。

　　五、全面补齐发展支撑短板，构建现代基础设施体系

　　完善综合交通大网络。完成遵义新舟机场改扩建，建成茅台机场和3个以上通用机场，组建通用航空公司，力争开通更多国际航线航班。建设铁路350公里以上、城市轨道交通63公里以上。新增高速公路750公里以上，改造提升国省干道2000公里、县乡道路640公里，硬化1.5万公里撤并建制村道路，建成4个中心城区综合客运枢纽、7个县级客运站以及一批多式零距离换乘交通枢纽。加快乌江、赤水河航运扩能和芙蓉江库区航运建设，水运航道突破1000公里，年通过能力达2500万吨以上，港口码头年吞吐能力突破2000万吨。

　　构建现代水利大体系。建设遵义观音大型水利枢纽工程，新建中型水库11座、小型水库34座以上，建成中型水库12座以上、小型水库22座以上，新增供水能力8亿立方米、年供水能力达36亿立方米以上，实现市有大型水库、县有中型水库、乡有稳定供水源。全面实施乡镇集镇农村人饮巩固工程。治理病险水库40座。抓好遵义、湄凤余、仁赤习大型灌区续建配套和节水改造，加快重点中小型灌区节水配套改造，新增、恢复、改善灌溉面积60万亩。基本解决工程性缺水问题，形成有雨能蓄、干旱能灌、洪涝能排的现代水利体系。

　　提升能源基础大保障。构建水火互济、清洁能源为补充的能源供给体系，新建风电项目52个，新增中小水电装机20万千瓦以上，电力总装机达1200万千瓦以上。建成500千伏变电站1座、220千伏变电站6座，新增220千伏及以上线路680公里，推进新一轮农村电网升级改造。加快“一主一环五支”（40）输气管道建设，新建天然气城际管网300公里，城市燃气综合普及率达95%，城乡（城市、乡镇及农村）燃气普及率达70%以上。

　　六、全面增强民生保障能力，构建现代公共服务体系

　　优先发展教育。深化基础教育综合改革，推动高中教育特色发展、职业教育产教融合、高等教育内涵式发展，积极发展学前教育、特殊教育、民族教育和继续教育，支持和规范民办教育，全面实现县域义务教育均衡发展，巩固提高“普十五”教育水平，大幅提升教育质量。优化城乡教育资源配置，新改（扩）建城市中小学40所以上。全力申建遵义大学、遵义医科大学，支持遵义医专申报本科层次高等院校、遵义体校申报遵义体育职业学院，建成贵州航天职院新蒲校区，提升遵义干部学院品牌影响力，遵义大学城师生规模达10万人。

　　促进就业创业。实施全民创业行动计划，健全覆盖城乡的公共创业就业服务体系，新增城镇就业55万人、转移农村劳动力45万人，城镇登记失业率稳控在4.2%以内。通过政策扶持、产业发展、劳务输出、技能培训等，促进易地搬迁群众、返乡农民工、高校毕业生、城镇困难人员、退伍复员军人等群体创业就业。全面提高就业服务质量，构建和谐劳动关系。

　　建设文化强市。深入践行社会主义核心价值观，传承红色基因，讲好遵义故事，构筑红色文化传承、公共文化服务、民族民间文化保护利用和“遵道行义·自强不息”城市精神“四大体系”，成功创建全国文明城市。实施“531”产业工程（41），推进文艺精品创作。促进“文化惠民”和文化消费，实现城乡公共文化服务设施全覆盖。力争把“遵义·长征文化产业园”创建为国家级文化产业示范园。大力支持茅台申遗、双河洞申建世界地质公园。

　　推动全民健身。加快公共体育设施建设，实现城市新建社区和乡镇、行政村体育设施全覆盖，推动公共体育设施和企事业单位、学校体育场地设施向社会开放，人人享有基本公共体育健身服务。建立全民健身管理资源库、服务资源库和公共服务信息平台，深入开展全民健身活动，组建国酒国球队等一批跻身国际国内顶级赛事的专业联队，坚持每两年举办一届市运会，高水平承办2018年贵州省第十届运动会，建设山地特色体育强市。

　　加强社会保障。全面落实全民参保计划，实施社会保障兜底工程，推进社保关系跨地区转移接续，适时并轨城乡居民基本医疗保险。改造城镇棚户区20万户以上、农村危房7.7万户。健全社会救助体系，加快社会福利、慈善、社会组织和残疾人、老年人事业发展。深化“三关爱”（42）工程。大力提高防灾减灾能力。

　　创新社会治理。深入实施安全发展战略，建立横到边、纵到底、零死角、无盲区监管网络，决不以牺牲人的生命为代价换取发展。巩固提升“平安遵义”建设水平，完善社会治安立体防控体系，提升特殊人群管理服务水平，创新群众工作方法，继续推广社会矛盾化解“余庆经验”（43），力争蝉联“长安杯”。抓好“全国双拥模范城”、民族团结进步等创建活动。深入开展“七五”普法，提高公共法律服务工作水平。

　　七、全面加强政府自身建设，构建现代政府治理体系

　　着力打造廉洁政府。深入贯彻党的十八届六中全会精神，自觉服从核心，坚决维护核心，全面落实从严治政新要求。严格执行民主集中制和行政首长负责制。坚持标本兼治、综合治理、惩防并举、注重预防方针，从严从实抓好党风廉政建设。坚决落实中央“八项规定”精神，坚决执行“约法三章”，坚决向“四风”问题宣战，筑牢“制度铁笼”“数据铁笼”，重拳整治不正之风。

　　着力打造法治政府。坚持法无授权不可为，法定职责必须为，自觉厉行法治、依法行政。加强法制审查、行政复议等工作，健全政府法律顾问、决策执行纠偏、政府失信责任倒查及追究、行政机关负责人出庭应诉等制度，严格兑现向社会及行政相对人作出的政策承诺。自觉接受市人大及其常委会法律和工作监督、市政协民主监督、新闻舆论和社会公众监督，加强行政监察和审计监督，保证政府一切工作都在法治的轨道上、人民的监督下规范运行。

　　着力打造高效政府。努力建高效率人民政府、当高效率公务人员，加大对新知识、新经验的学习应用，提升研究新问题、解决新难题的能力，不断提高政策连续性和含金量，增强群众获得感和幸福感。大力推进“云上遵义”建设，提升政务服务水平，坚决砍掉各类无谓证明和繁琐手续，全心全意为人民服务，千方百计为群众解难。坚持一张蓝图干到底，增强弱鸟先飞意识，发扬滴水穿石精神，践行马上就办作风，加大督查督办力度，决不在遵义今天的长征路上落伍掉队，坚决完成时代赋予的历史使命，坚决兑现向全市人民许下的庄严承诺。

　　2017年主要目标任务

　　今年是实施“十三五”规划的重要一年和推进供给侧结构性改革的深化之年，机遇与挑战并存，信心比黄金重要。我们要深入贯彻“五大新发展”理念，坚持稳中求进工作总基调，以推进供给侧结构性改革为主线，全力以赴稳增长、促改革、调结构、惠民生、防风险，以优异成绩迎接党的十九大和省第十二次党代会胜利召开。

　　主要预期目标是：地区生产总值增长12.5%，规模工业增加值增长12%，500万元以上固定资产投资增长20%以上，财政总收入、一般公共预算收入分别增长10%，社会消费品零售总额增长12.5%，金融机构存贷款余额分别增长25%、20%，城乡居民人均可支配收入分别增长10%、11%，全面小康实现程度达92.5%，增比进位综合测评保持在全省第一方阵；居民消费价格指数、节能减排降碳等指标控制在省调控目标内。

　　围绕以上目标，我们将在以下九个方面更加聚力：

　　一、更加聚力推进精准脱贫。确保减少贫困人口10万人，贫困县农村居民人均可支配收入达9000元以上，习水县和318个贫困村按国标脱贫退出，少数民族和民族地区48个贫困村2.8万人稳定脱贫。全力打好易地扶贫搬迁决胜之仗，如期完成“十三五”搬迁任务。集中力量抓好产业扶贫，大力开展贫困村“一村一品”产业推进行动，用足用好脱贫攻坚产业扶贫基金和精准扶贫“特惠贷”，因地制宜发展蔬菜、干鲜果、花卉苗木、生态畜牧等产业，农村电子商务交易额增长30%以上，建成58个农旅一体化示范点。统筹推进上海对口帮扶、教育医疗和社会兜底脱贫等各项工作，抓好同步小康驻村帮扶、“百企联村・千企帮户”和“城市解困・和弦行动”（44），打造147个脱贫攻坚示范点。大力宣传“贫穷不可怕，可怕的是懒惰”，引导全社会向脱贫攻坚先进典型学习，不断增强群众内生动力。

　　二、更加聚力促进产业转型。扎实开展质量品牌提升行动，培育中国驰名商标1个、贵州著名商标20个、贵州名牌产品30个。对全市所有白酒企业进行全面调查摸底，制定支持白酒产业健康发展的意见，精心办好茅台镇酒文化节。推进茶及茶产品精深加工，创建“遵义绿”品牌，提升“遵义红”影响力。建成具有国际品牌竞争力的精品酒庄、精品茶庄20个以上。完成遵义卷烟厂异地技改主体工程建设和天义电梯整体搬迁，建成遵义铝业15万吨填平补齐项目和航天精工紧固件、凯星液力变速器等重点项目，加快务川氧化铝、绥阳煤电锰一体化、赤水30万吨生活原纸和纸制品等重大项目建设。深入推进农业供给侧结构性改革，加快现代高效农业园区建设，大力调减玉米种植，推动土地流转，新增家庭农场500家、市级农业龙头企业50家，认定无公害农产品产地面积100万亩、绿色食品企业20家、有机农产品30个以上、地理标志保护农产品2个以上、生态原产地保护产品5个，建成赤水国家级出口农产品质量安全示范区，实现农产品质量安全检验检测全覆盖，完成规模农产品加工业产值380亿元。

　　三、更加聚力培育新兴产业。全力推进大数据“聚通用”，聚集高端智能终端产品及配套生产企业30家以上，实现大数据企业主营业务收入380亿元、增长20%。加快新能源汽车产业园建设，建成容百10万吨锂电正极材料等项目，新能源汽车生产及配套充电设备有新突破。推动正安页岩气进入实质性开发。建成一批风电、水电项目。办好全市旅发大会，建成茶海自行车公路、200公里生态旅游步道和一批长征体验项目，确保游客接待量突破1亿人次、旅游综合收入突破1000亿元，分别增长36%以上，申报国际旅行社2家以上。在中心城区启动3个以上五星级酒店和3个以上温泉综合体建设。加快黔北现代物流新城、新蒲金融商务中心、虾子辣椒城等重点项目建设。规划建设中心城区快递物流园和县城快递物流集中仓储点，完成电子商务交易额513亿元。建成国家级、省级和区域性检验检测中心13家。建成唯一国际、奥特莱斯等大型商业综合体，新（续）建大型市场20个以上，升级改造县乡农贸市场20个以上。

　　四、更加聚力扩大项目投资。实施市级重大工程项目808个，新开工300个以上、建成投产110个以上。建成园区污水处理厂7座、标准厂房200万平方米以上，开工建设投资3000万元以上产业项目400个，建成投产130个以上。加快遵龙大道建设，建成凤新快线、长大立交等骨干交通，加快天义厂、原遵义电厂等城中村、城郊结合部及县城、集镇棚户区改造，户籍人口和常住人口城镇化率分别提高到41%、49%。推进新型城镇化试点、整县推进小城镇试点和“小康寨”整县推进示范县建设，加快“六型”示范小城镇建设，打造100个“四在农家・美丽乡村”示范点。扎实抓好“十大工程”建设，彻底整治城市管理乱象，确保成功创建全国文明城市。建成渝黔快铁和川黔铁路遵义城区段外迁工程；启动遵义新舟机场改扩建二期工程，茅台机场实现通航；确保江习古高速（遵义段）建成通车，加快兰海高速贵遵扩容、遵绥延伸线等高速公路建设，确保4条、力争6条高速公路新开工；建成国省干线278公里；力争建成投用构皮滩电站过船设施，启动遵义港漩塘港区进港公路和码头建设。实现9座水库下闸蓄水，完成8座水库大坝建设，新开工10座骨干水源工程，力争开工观音大型水库；启动湄江河湄潭县城河段分洪工程建设。投产500千伏变电站1座、220千伏变电站2座、110千伏变电站1座，加强高压网架、中心村（小城镇）电网升级改造和易地扶贫搬迁点、产业园区供电配套。开工建设习水—正安—务川、遵义—绥阳天然气管道。建设通信基站1500个，实现全市移动通讯信号、4G网络深度覆盖和村村通光纤。

　　五、更加聚力壮大县域经济。新增市场主体6万户以上，新增规模工业企业150户以上、限上商业企业100户以上。深入开展“民营企业服务年”活动，民营经济比重提高到56%。力争至少1家企业在主板、中小板、创业板或境外资本市场上市，在新三板挂牌10家、力争20家。力争引进世界500强企业5家，招商引资到位资金增长15%。落实更加精准的差异化指导和考核措施，确保经济总量中部增长13%、西部增长13%、东部增长14%、北部增长14.5%。加大小康创建力度，确保习水县、桐梓县、湄潭县、绥阳县全面小康创建达标，余庆县通过省级同步小康达标验收。

　　六、更加聚力守牢生态底线。建成乡镇污水处理厂56座，城镇污水处理率达90%，城乡生活垃圾无害化处理率达90%，一般工业固废综合利用率达65%，中心城区环境空气质量优良率达92%，重要水功能区水质达标率达85%，县城以上集中式饮用水源地水质达标率保持100%。抓好赤水市、习水县国家生态文明示范工程试点，推进赤水河流域生态经济示范区、洛安江流域生态文明先行示范区建设，力争创建2个国家级、3个省级生态文明建设示范县。实施营造林75万亩，森林覆盖率提高到58%以上。治理水土流失面积70平方公里、石漠化面积72平方公里，治理中小河流14条。抓好“生态日”活动，全面推进绿色学校、机关、村寨、社区、家庭创建活动。

　　七、更加聚力推动改革开放。狠抓中央和省、市重大改革事项落实，大力推进基础性关键性领域改革，在对外开放、生态文明、脱贫攻坚、行政审批、国有企业、产权制度、电力体制、资源配置、农村产权、财税金融、社会事业、依法治市等领域，集中推出一批力度大、措施实、接地气的改革措施。力争遵义综合保税区、国家高新区获批，推动遵义国家经济技术开发区扩区移位，遵义新舟机场航空口岸申建取得重大突破，遵义软件园、大学生创业园建设取得实质性进展。精心办好“茶博会”“辣博会”和正安首届国际吉他艺术节等活动。新增外资企业10家、外贸企业50家，外贸进出口总额和实际利用外资分别增长20%。组建产业技术创新战略联盟，培育省级以上科技型企业50家，申请发明专利1100件以上，高新技术产值增长25%，引进省外优秀科技领军人才5—10人。

　　八、更加聚力防控风险隐患。创新财政资金使用方式，加强预算绩效管理，严格规范政府举债，推进市场化债转股、投贷联动、股权融资创新，强化国有企业管理，积极帮助民营企业、中小企业解决融资难融资贵等问题，严厉打击非法集资，确保不发生以县为单位的财政金融风险。严格落实安全生产责任，坚决遏制重特大安全生产事故发生。加强城市安全监管和地质灾害防治，加大防范和打击“两违”（45）力度。加强社会治安综合治理，抓好“天网工程”建设和运行维护，依法严厉打击各类违法犯罪。强化重大事项社会稳定风险评估，健全基层社会服务管理体系。妥善解决中心城区房屋所有权登记历史遗留问题。全面推行大数据、网格化服务管理，及时排查化解各类社会风险，确保人民群众安全感和满意度保持在全省前列。

　　九、更加聚力办好民生实事。易地扶贫搬迁2.5万户10.2万人，确保每户搬迁贫困户至少有1人稳定就业。完成学前教育项目120个、“全面改薄”（46）项目100个，新（改、扩）建中心城区学校11所，确保6个县（市、区）通过县域义务教育基本均衡国家认定，建成市青少年示范性综合实践基地。实现全市乡镇卫生院远程医疗全覆盖。新增城镇就业12.6万人，转移农村劳动力10.5万人，城镇登记失业率控制在4.2%以内。实施城镇棚户区及城市危房改造10万套，改造农村危房4万户，改善25个传统村落人居环境。建成市文化馆、奥体中心、体育运动学校、水上运动训练基地，推动大型文化、体育场馆免费或低收费开放。建成农村公路5100公里，建设便民码头3个，解决最后46万农村群众饮水安全问题。在中心城区建成10个山体公园、18个湿地公园。完成中心城区剩余未改造小微循环道路和100个社区最后65公里排污管网改造任务，全市新增公共停车位4000个。建设4套中心城区环境噪声自动监测屏，实现乌江流域乡镇污水处理设施全覆盖。

　　各位代表！全力摆脱贫困战鼓齐鸣，决胜全面小康号角嘹亮，打造西部内陆开放新高地旌旗高扬。让我们更加紧密地团结在以习近平同志为核心的党中央周围，在市委的正确领导下，因势而谋、因势而动、因势而进，为夺取第一个百年目标伟大胜利、创造更加幸福美好未来而努力奋斗！
